# The effects of Eculizumab on the pathology of malignant atrophic papulosis

**DOI:** 10.1186/1750-1172-8-185

**Published:** 2013-11-26

**Authors:** Cynthia M Magro, Xuan Wang, Francine Garrett-Bakelman, Jeffrey Laurence, Lee S Shapiro, Maria T DeSancho

**Affiliations:** 1 Department of Pathology and Laboratory Medicine, Weill Medical College of Cornell University, Box 58, Room F-309, 1300 York Avenue, New York, New York 10065, USA; 2 Division of Hematology/Medical Oncology, Department of Medicine, Weill Medical College of Cornell University, New York, New York 10065, USA; 3 The Center for Rheumatology, LLP, 1367 Washington Ave., Suite 101, Albany, NY 12206, USA

**Keywords:** Eculizumab, Degos disease, Complement, C3d, C5b-9, Caspase 3

## Abstract

**Background:**

Degos disease is a frequently fatal and incurable occlusive vasculopathy most commonly affecting the skin, gastrointestinal tract and brain. Vascular C5b-9 deposition and a type I interferon (IFN) rich microenvironment are held to be pathogenetically important in the evolution of the vascular changes. We recently discovered the use of eculizumab as a salvage drug in the treatment of near fatal Malignant atrophic papulosis (MAP). The effects of eculizumab on the pathology of MAP are explored.

**Methods:**

Archival skin and gastrointestinal biopsy material was procured over a 2.5-year period before and after eculizumab therapy in our index case. Routine light microscopy and immunohistochemical assessment for C3d, C4d, C5b-9, MxA and caspase 3 were examined. Direct immunofluorescent studies were also conducted on select biopsy material.

**Results:**

The patient had received eculizumab as a emergent life saving measure and following rapid improvement he continued with biweekly infusions for 4 years. Although improved he continues to have signs and symptoms of persistent abdominal disease. Pre-Eculizumab biopsies showed an active thrombotic microangiopathy associated with a high type I interferon signature and extensive vascular deposits of C5b-9 in skin and gastrointestinal biopsies. Endothelial cell apoptosis as revealed by Caspase 3 expression was noted. Inflammation comprising lymphocytes and macrophages along with mesenchymal mucin was observed as well. Post-eculizumab biopsies did not show active luminal thrombosis but only chronic sequelae of prior episodes of vascular injury. There was no discernible caspase 3 expression. After 12 months of therapy, C5b-9 was no longer detectable in tissue. The high type I IFN signature and inflammation along with mucin deposition was not altered by the drug. In addition, there was little effect of the drug on the occlusive fibrointimal arteriopathy which appears to be one characterized by extensive myofibroblastic expansion of the intima potentially as revealed by staining for smooth muscle actin without immunoreactivity for desmin and myogenin.

**Conclusions:**

Complement activation and enhanced endothelial cell apoptosis play an important role in the thrombotic complications of MAP. However, the larger vessel proliferative intimal changes appear to be independent of complement activation and may be on the basis of other upstream mechanisms. Monitoring C5b-9 deposition in tissue is likely not of great value in assessing treatment response to eculizumab given the persistence of C5b-9 in tissue for several months despite clinically effective C5 blocking therapy. A more integrated approach addressing upstream and downstream pathways in addition to those attributable to complement activation are critical for the successful treatment of MAP. Eculizumab may be used as salvage therapy in critically ill patients with thrombotic microangiopathy.

## Background

Malignant atrophic papulosis (MAP) falls under the alternative appellation of Degos disease and Kohlmeier-Degos disease [1-3]. It is a severe and frequently progressive angiopathy syndrome targeting certain organs, most commonly the skin, gastrointestinal tract, and central nervous system, although other organ sites including the lung and heart can be involved [4-6]. This syndromic complex has very distinctive cutaneous lesions characterized by depressed porcelain plaques with an atrophic center. Similar lesions can affect the gastrointestinal tract as well.

There are two fundamental components to the vascular disease in MAP, namely a thrombotic microangiopathy targeting capillaries and venules, and a strangulating fibrointimal arteriopathy involving small and medium-sized arteries [7]. The thrombotic microangiopathy affecting capillaries and venules is most commonly manifested in the skin and is the pathogenetic basis of the characteristic cutaneous porcelain plaques. Nevertheless, a thrombotic microangiopathy can affect any organ system and produce acute ischemic symptoms analogous to other catastrophic thrombotic microangiopathy syndromes such as antiphospholipid antibody syndrome, thrombotic thrombocytopenic purpura and hemolytic uremic syndrome [8,9]. In contradistinction, a significant component of the gastrointestinal pathology is attributable to the larger vessel fibrointimal arteriopathy of the submucosal vessels and serosa. Similar arteriopathic changes affect other organs, most notably the subdural arteries and coronary vasculature.

We have shown in previous studies that there is a role for both type I interferons as well as C5b-9 in the evolution of the microangiopathy and larger vessel arteriopathic changes that define MAP [7]. Human Myxovirus resistance protein 1 (MxA), a widely recognized and accepted marker of type I interferon bioactivity, is highly expressed in MAP, and its expression parallels the pattern of C5b-9 deposition [7]. Due to the extent of membranolytic attack complex deposition within the cutaneous vasculature, we previously hypothesized that blocking C5 through the administration of eculizumab could potentially halt disease progression and possibly even offer a cure to this once fatal disease. We discovered that the drug has a beneficial effect in catastrophic presentations of MAP. However, patients continued to experience symptoms related to the disease, especially in regards to gastrointestinal complications, indicative that the blocking of C5b-9, while significantly beneficial and potentially life saving, does not appear to be curative of MAP.

The purpose of this study is to better understand the role of complement and, more specifically, C5b-9 (i.e. the membranolytic attack complex) in the pathogenesis of MAP. It is important to delineate more precisely the sequelae of C5 blockage on the pathology of Degos disease to appreciate both the beneficial effects and inherent limitations of the drug. We had the opportunity to study multiple skin and gastrointestinal tissue samples prior to the administration of eculizumab and for up to two and a half years after the commencement of drug in one patient.

## Methods

Biopsy material was available from a patient with MAP who has been receiving biweekly infusions of eculizumab since October 2009. The tissues studied included skin, small and large intestines, and pericardium. Specimens available for assessment included those before and after eculizumab therapy spanning a time period of July 2009 to December 2011. In several cases, additional tissue was available for immunofluorescent studies. The tissue was analyzed with routine light microscopy and immunohistochemical stains were performed to assess for deposition of C5b-9, C3d, and C4d in tissue. In addition, the expression of MxA was explored given the role of type I interferons in the pathogenesis of the microangiopathy. To better characterize the nature of the proliferative intimal response, smooth muscle actin, desmin, myogenin, CD34, and CD14 iimunohistochemical stains were conducted on gastrointestinal resection specimens.

Immunohistochemical staining was performed on 4-μm-thick, formalin-fixed, paraffin-embedded tissue sections by using the Bond 3 Autostainer (Leica Microsystems, Buffalo Grove, IL) and the Bond Polymer Refine detection system (Leica Microsystems). Cleaved Caspase-3 (#9661, Cell Signaling Technology, Boston, MA) was used at a dilution of 1:50. C5b-9 (clone aE11, Dako, Carpinteria, CA) was used at a dilution of 1:250. Antigen retrieval was performed for Caspase-3 with Bond Epitope Retrieval Solution no. 1 (Leica Microsystems). Antigen retrieval for C5b-9 was performed using Enzyme 1 from the Bond Enzyme Pretreatment Kit (Leica Microsystems). Immunostaining was performed according to a modified manufacturer’s protocol and sections were counterstained with hematoxylin, dehydrated, and embedded in Cytoseal XYL (Thermo Scientific, Kalamazoo, MI).

Immunofluorescent studies were conducted on fresh frozen tissue of the skin and gastrointestinal tract on selected cases according to previously published protocols [6].

## Results

### Brief clinical synopsis

46-year-old male who initially presented with intermittent abdominal cramping and non-bloody diarrhea in 2006. In 2007, he started to develop macule- and plaque-like lesions over his upper extremities and trunk. Despite a negative lupus workup, he was given hydroxychloroquine with transient mild subjective improvement in his abdominal symptoms but no change in his skin lesions.

In July 2009, he developed worsening bilious emesis with fever and abdominal pain of several days’ duration and presented to our hospital. On a chest X-ray, he was found to have sub-diaphragmatic free air as well as pleural effusions. He was emergently sent to the operating room (OR) for exploration. During surgery, he was found to have scattered depressed whitish plaques on his colon and small intestines, mirroring the gross morphology of the skin lesions. A perforation was found in the terminal ileum, which was resected. During this hospital stay, his physical examination also revealed numerous hyperpigmented macules with central atrophy on the trunk and extremities. A few erythematous papules with scaling in the center on the neck and shoulders were also noted. Skin biopsies of these lesions showed findings consistent with Degos disease. The pathological features of the resected small intestine and skin biopsies were discussed below as pre-eculizumab changes. Post-surgery, the patient received intravenous immunoglobulin (IVIg) and was discharged home on prohylactic-dose of low molecular weight heparin.

After discharge, he still experienced recurrent intermittent episodes of abdominal pain. In September 2009, he suffered another episode of severe abdominal pain He was diagnosed of a partial small bowel obstruction. He went to the OR and had lysis of adhesions. During surgery, that his small intestine plaques related to MAP seen at the first surgery appeared unchanged in number and distribution.

In October 2009, he was admitted to the medical intensive care unit (MICU) with left pleural and pericardial effusions complicated by cardiac tamponade. A thoracotomy performed with pericardial window chest tube placement. A concomitant pericardium biopsy showed changes consistent with MAP, the details of which are discussed below as pre-Eculizumab pericardium changes. His hospital stay was further complicated by bilateral subsegmental pulmonary emboli and biventricular failure with a left ventricular ejection fraction (LVEF) of 17%. This rapid decline in his status prompted the decision to initiate eculizumab treatment, with simultaneous support care including Dobutamin, Caspofungin for candida infection and Valganciclovir for his cytomegaloviremia. He experienced significant clinical improvement within 2–3 days of initiation of treatment, and his LVEF increased to 57%. After receiving the initial 3 cycles of Eculizumab, the patient was discharged home.

He continued eculizumab treatment as an outpatient without complications. His symptoms remained stable and he reported fewer new skin lesions in February 2010, the patient was hospitalized again for small bowel obstruction and lysis of adhesions. The ostomy was resected and the pathological findings are discussed below as post-eculizumab changes. Post surgery, he was found to have peritonitis with proteinaceous ascites, and small bowel wall thickening on imaging. A repeat skin biopsy was performed in March 2010, which showed no significant active endothelial cell necrosis. The findings of this and additional follow-up skin biopsies are detailed below as post-eculizumab changes. The patient’s symptoms again resolved with treatment and he was managed as an outpatient with biweekly infusions of Eculizumab.

In July 2011, he developed walled-off sigmoid colon perforation unresponsive to medical therapy, for which he underwent exploratory laparotomy with sigmoid colon resection, mobilization of splenic flexure and diverting ileostomy. Examination of the colon showed evidence of remote perforation, and fibro-obliterative vascular changes although without thrombosis and active vasculitis. The results are discussed in details below as the gastrointestinal findings post-Eculizumab, along with results of the concomitant skin biopsies.

By the end of 2011, the patient had received 61 cycles of eculizumab without complications, and had been largely clinically stable. He continued to experience abdominal pain; however, the pain is not severe and unaccompanied by nausea or vomiting. His skin exam shows a few healed scars along with scattered erythematous papules. However the active inflammatory lesions have not undergo evolution into an atrophic porcelain white macule. In the last year however he has had a progressively wasting course due to persistent and progressive gastrointestinal disease.

#### Histologic results

We will review the biopsies sequentially and consider two time points: before and after Eculizumab therapy.

#### Skin

**Figure 1:**
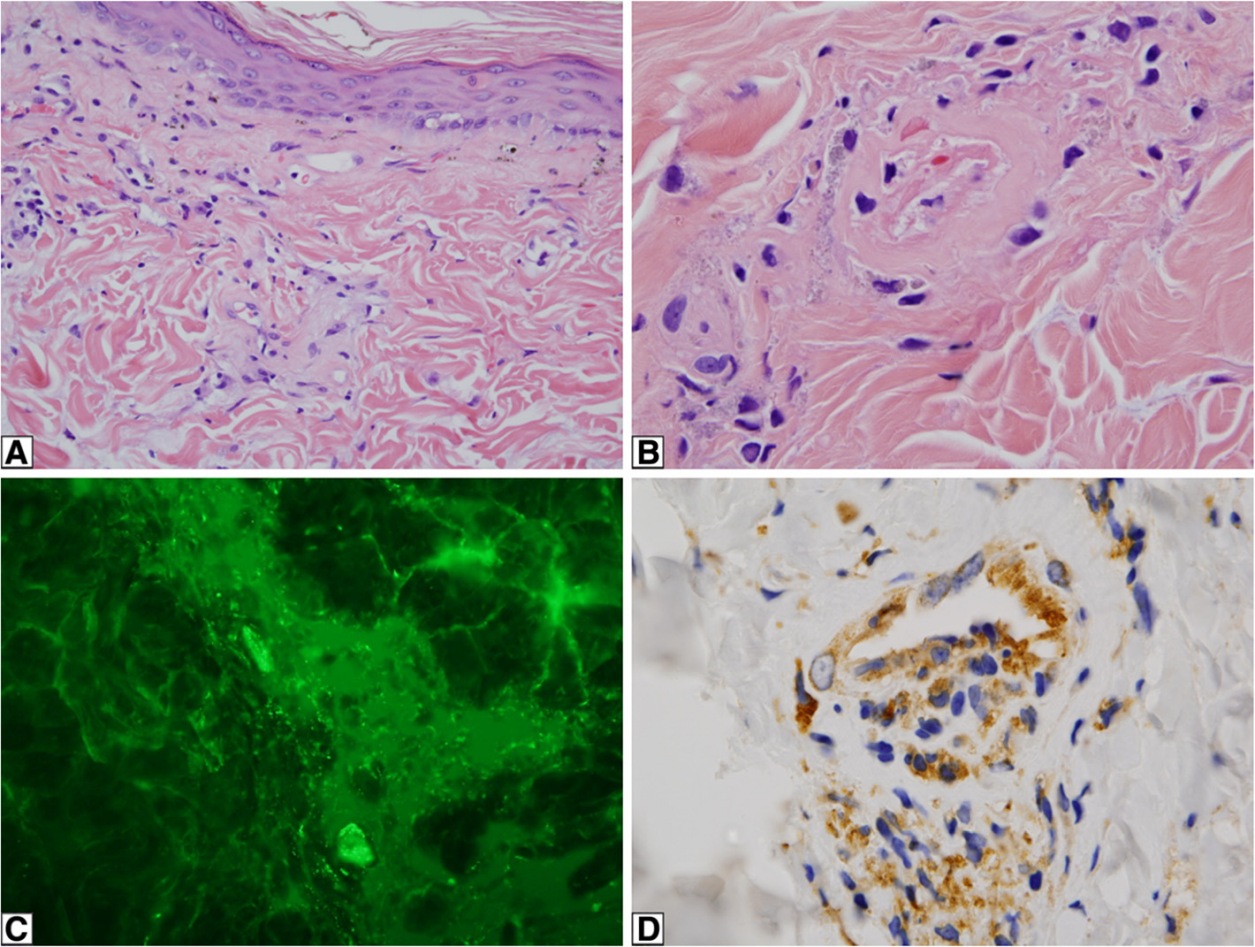
**A, B, C, and D: Skin histology pre-Eculizumab microvascular changes along with the pattern and extent of C5b-9 deposition and MXA expression in tissue.** An erythematous lesion biopsied in July 2009 shows a thrombotic microangiopathy along with dermal chronic microvascular changes characterized by thickened basement membrane zones along with superficial vascular ectasia and foci of vascular drop out. The chronic microvascular changes are present in both the **A** (200x) and **B** (1000x) biopsies while intraluminal fibrin deposition is noted in **B**. (hematoxylin and eosin) In Figure 1A, there is a sparse perivascular lymphocytic infiltrate. The vessels appear hyalinized attributable to vascular basement membrane zone reduplication. In Figure 1B, the vessel is largely devoid of endothelium and contains a loosely adherent thrombus. The vessel is also noticeably thickened. Figure 1C exhibits the pattern of C5b-9 deposition pre-eculizumab. There is prominent deposition of C5b-9 by immunofluroescence in the vessels in the pre-eculizumab biopsy (400x). Figure 1D demonstrates MxA in skin biopsy pre-Eculizumab. There is extensive staining for MxA in the epidermis, endothelium and inflammatory cells in the pre-eculizumab biopsy indicative of a type I IFN rich microenvironment (diaminobenzidene, 1000x).

Prior to eculizumab administration: An erythematous lesion biopsied in July 2009 showed a thrombotic microangiopathy with foci of vascular drop out (Figure 1A, 1B). A scarred lesion was biopsied a few days later showed fibrosis, epidermal thinning and vascular drop out. In both biopsies prominent vascular deposition of C5b-9 (Figure 1C) was noted along with extensive immunoreactant deposition for MXA, the latter showing localization in the epidermis, endothelium and inflammatory cells (Figure 1D). There was focal nuclear staining of caspase 3 in the endothelium.

**Figure 2:**
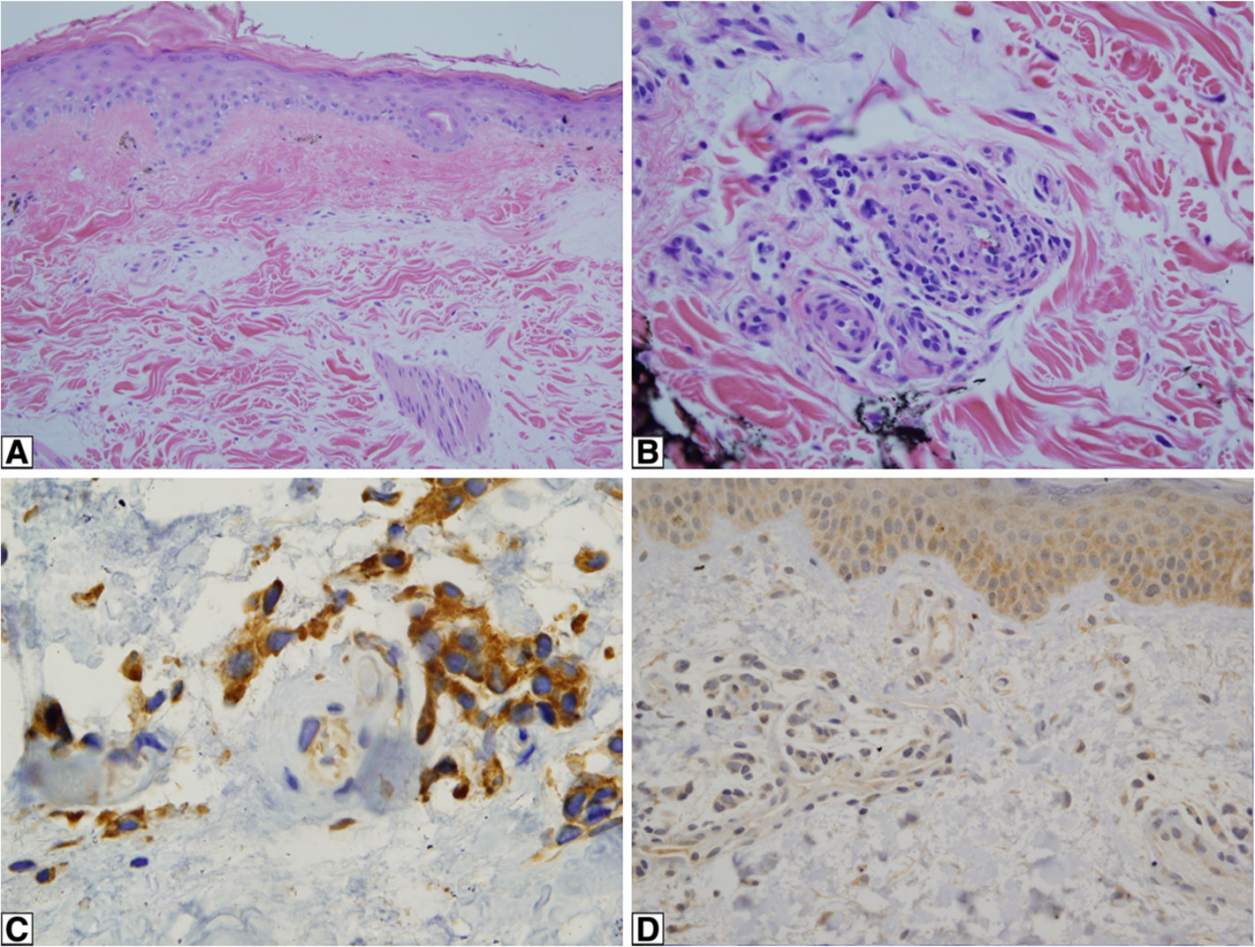
**Skin microvascular changes and immunohistochemical assessment for C5b-9 and MXA staining post-eculizumab. A**. Skin histology post-eculizumab. Subsequent to receiving Eculizumab, the patient underwent 6 sequential biopsies 2 weeks to 25 months after commencing the drug. In none of the post-eculizumab biopsies is there evidence of active endothelial cell injury and vascular thrombosis. A common finding in all of the biopsies is one reflective of antecedent episodes of microvascular injury characterized by subepidemal fibrosis with vascular drop out, vascular basement membrane zone duplication and vascular ectasia (diaminobenzidene, 200x). **B**. Inflammatory cell infiltrate in skin biopsy post-eculizumab. A variable inflammatory cell infiltrate comprising lymphocytes and histiocytes primarily arranged around blood vessels is present in all of the biopsies. The biopsy procured in March 2011, 16 months after the commencement of eculizumab, shows a striking lymphocytic and histiocytic infiltrate with abundant mucin deposition. Despite the exuberant inflammation, active microvascular injury is not seen. In particular discernible vascular thrombosis is not observed (hematoxylin and eosin, 400x). **C**. MxA in skin biopsy post-eculizumab. In all post-Eculizumab biopsies, there continues to be prominent expression of MxA in the epidermis, inflammatory cells and endothelium (diaminobenzidene, 1000x). **D**. C5b-9 in skin biopsy post-eculizumab. Nine months after the commencement of the drug, C5b-9 was no longer apparent (diaminobenzidene, 400x).

Post Eculizumab administration. Subsequent to receiving eculizumab, the patient underwent 6 sequential skin biopsies 2 weeks to 25 months after commencing the drug. In none of the post-eculizumab biopsies was there vascular thrombosis (Figure 2A) but only changes reflective of antecedent microvascular injury including subepidemal fibrosis, vascular drop out, vascular basement membrane zone duplication and vascular ectasia (Figure 2A). A variable perivascular mononuclear inflammatory cell infiltrate along with mucin deposition was present in post treatmentbiopsies (Figure 2B). High levels of MxA expression similar to pretreatment biopsies was noted (Figure 2C). C5b-9 both immunohistochemically and by immunofluorescence was present for 8 months after commencing the drug. Nine months after the commencement of the drug, C5b-9 was no longer apparent (Figure 2D). The caspase 3 stains in all biopsies examined were essentially negative.

#### Gastrointestinal specimens

**Figure 3:**
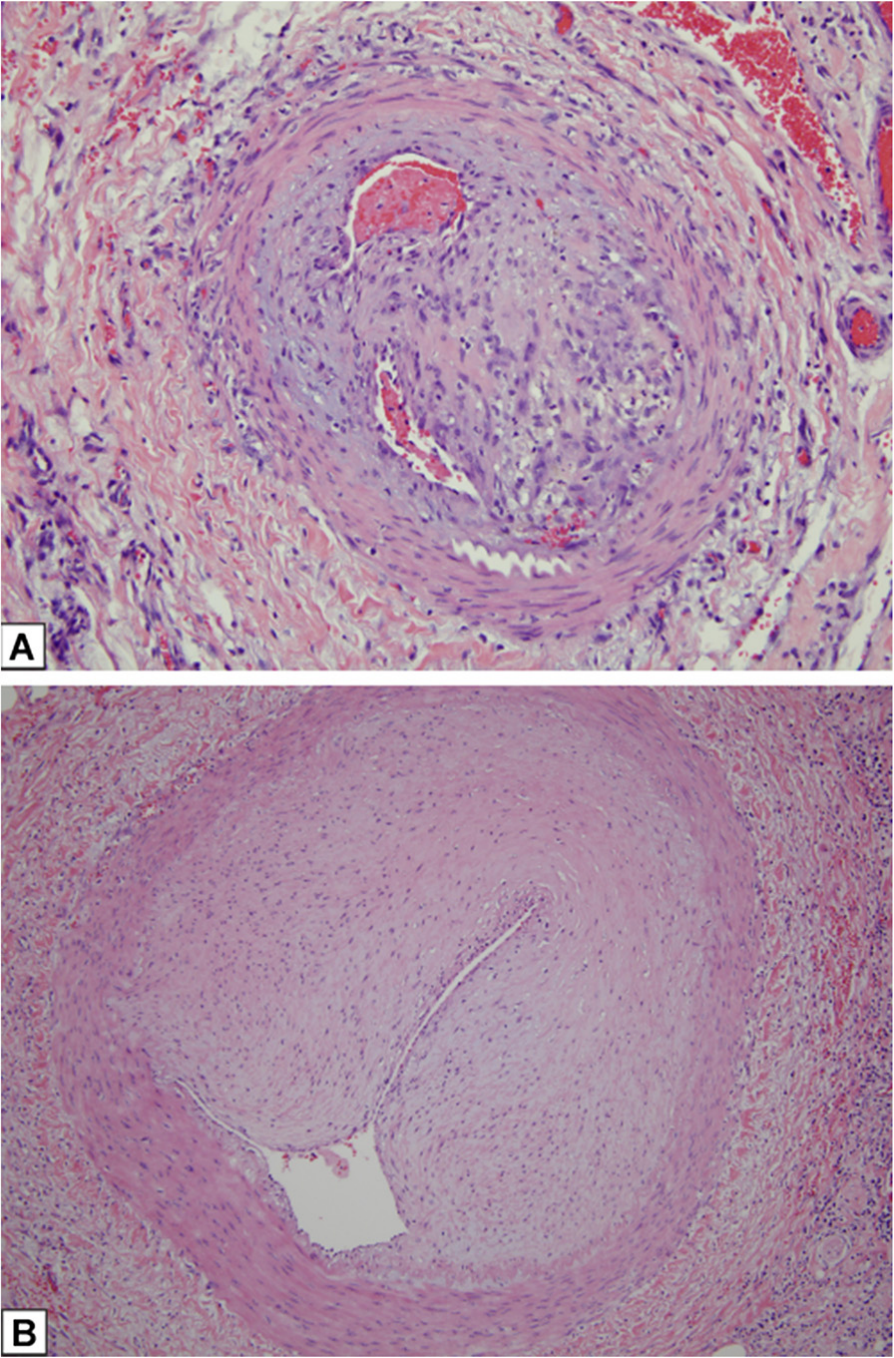
**A and B: Submucosal vessels of intestinal resection specimens pre- and post-eculizumab, respectively.** There were a total of 3 intestinal resection specimens available for assessment. One was obtained prior to the commencement of the drug (July 2009) and two were obtained 4 months (February 2010) and 21 months after starting the drug (July 2011). There are aspects of the pathology that was remarkably similar between the pre-eculizumab specimen and the one obtained 2 years after starting therapy. A striking fibro-obliterative arteriopathy affects small and medium-sized vessels of the submucosa as well as mesentery in both the pre- **(A)** and post-treatment **(B)** resection specimens (3**A**, hematoxylin and eosin, 200x, 3**B** hematoxylin and eosin, 200x). In **B**, the intimal proliferative changes are very striking comprising hypercellularity of the intima along with an increase in intimal matrix represented by collagen and hyaluronic acid.

**Figure 4:**
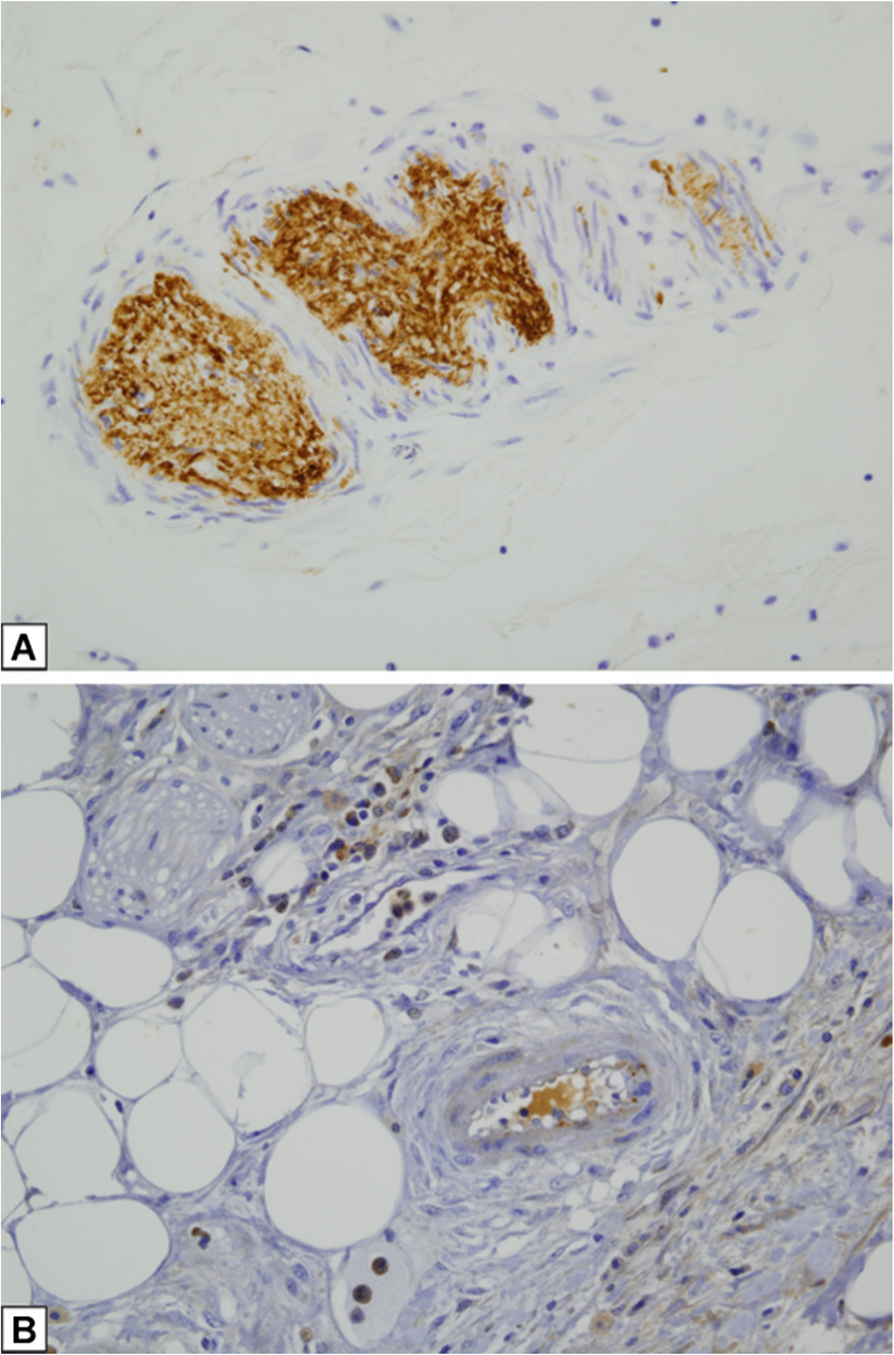
**A and B: C5b-9 in submucosal vessels of intestinal resection specimens pre- and post-eculizumab, respectively.** Immunohistochemical assessment reveals marked deposition of C5b-9 in vessels of the submucosa in the pre-eculizumab specimen (**A**, diaminobenzidene, 400x) while the intestinal resection specimen procured 21 months after commencing therapy (**B**, diaminobenzidene 400x) is devoid of vascular staining apart from nonspecific staining within the elastic tissue of blood vessels.

There were a total of 3 intestinal resection specimens available for assessment. One was obtained prior to the commencement of the drug (July 2009) and two were procured 4 months (February 2010) and 21 months after starting the drug (July 2011). There were aspects of the pathology that was remarkably similar between the pre-eculizumab specimen and the one obtained 21 months after starting therapy. In both there was a striking organizing acute and chronic serositis compatible with a recent perforation. An arteriopathy affected small and medium-sized vessels of the submucosa and mesentery (Figure 3A and 3B respectively). In particular the vessels showed a mucinous fibrointimal expansion and occlusion by fibrous collagenous thrombi. The intima was focally infiltrated by macrophages There was extensive mesenchymal mucin deposition within the submucosa accompanied by a lymphocytic infiltrate Focal staining of endothelial cells and interstitial cells (i.e. fibroblasts and inflammatory cells) for caspase 3 in the pre-eculizumab resection specimens was observed although the caspase 3 stains following initiation of eculizumab were negative. There was intense staining of endothelium, smooth muscle cells, stromal cells and inflammatory cells for MxA in the before and after eculizumab resection specimens. Immunohistochemical assessment revealed marked deposition of C5b-9 in vessels of the submucosa in the pre-eculizumab specimen while the intestinal resection specimen procured 21 months after commencing therapy was devoid of endothelial and intimal vascular staining (Figure 4A and 4B).

The resection specimen obtained 4 months after commencing the drug showed an obliterative fibrointimal arteriopathy without active vascular thrombosis C5b-9 were still detectable in tissue both by immunofluorescent as well as immunohistochemical assessment 4 months after commencing the drug although at 5 months post treatment an endoscopic biopsy was devoid of vascular C5b-9 deposition.

#### Phenotypic assessment of the fibro-obliterative arteriopathy

**Figure 5:**
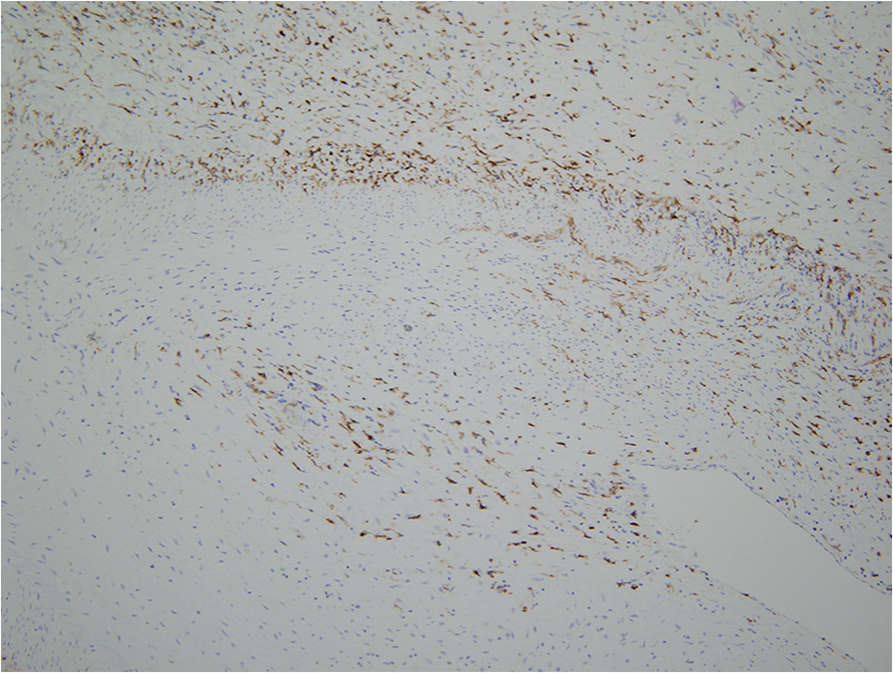
**The CD14 stain shows a striking influx of CD14+ monocytes throughout the gastrointestinal tract with margination of monocytes to lie in apposition to the adventitia.** Note the infiltration through the medial wall and extension into the intima (diaminobenzidene, 200x).

**Figure 6:**
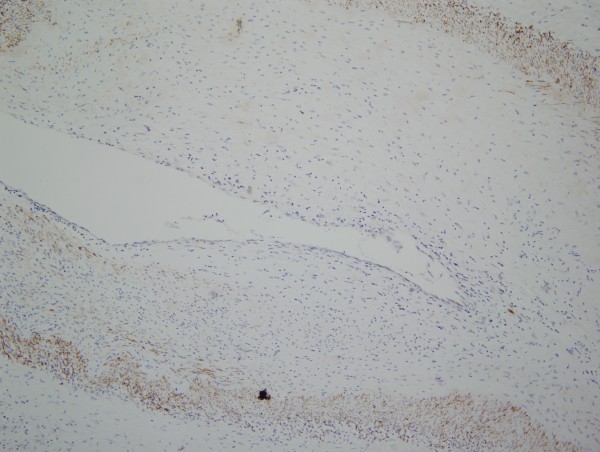
While the desmin extensively highlighted the medial wall smooth muscle cells, the hyperplastic intima is essentially negative indicative that the intimal cells are not of true medial wall smooth muscle origin (diaminobenzidene, 200x).

**Figure 7:**
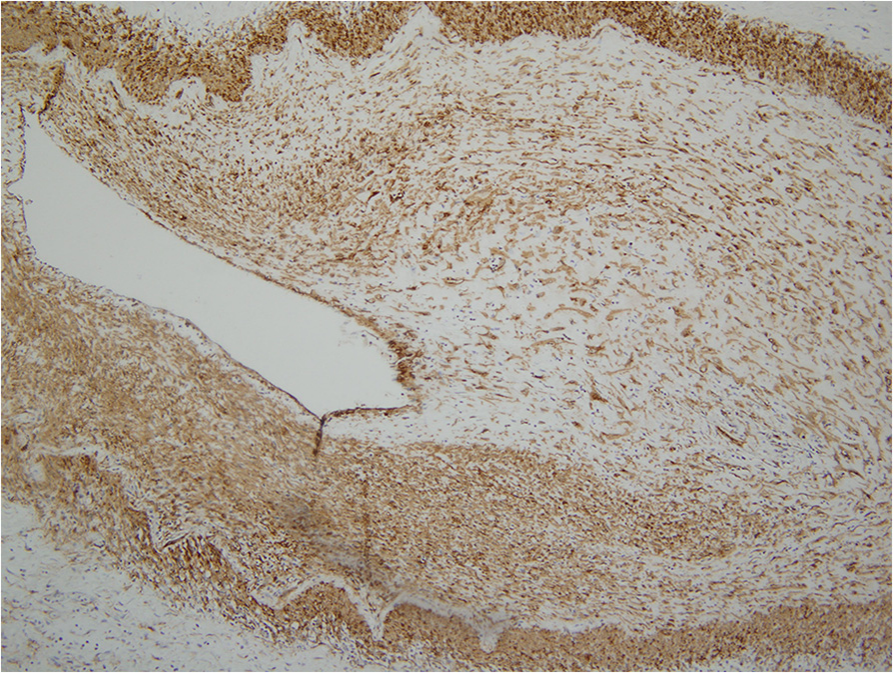
In this section of artery, there is very extensive staining for smooth muscle actin, an immunophenotypic profile that is common in myofibroblastic cells of stem cell hematopoietic and monocyte origin (diaminobenzidene, 200x).

Given the morphologic similarity between the arteriopathic changes in the pre-eculizumab specimen compared to the post eculizumab specimens, we conducted phenotypic studies only on the post eculizumab gastrointestinal resection specimen. The CD14 stain showed a striking influx of CD14+ monocytes throughout the gastrointestinal tract (Figure 5). In examining the diseased arteries, there was a margination of monocytes to lie in apposition to the adventitia with infiltration through the medial wall and extending into the intima; the cells exhibited a spindled morphology (Figure 6). While the desmin extensively highlighted the medial wall smooth muscle cells, the hyperplastic intima was essentially negative (Figure 7) A similar result was seen with myogenin. Very extensive staining was noted for smooth muscle actin. Staining was not observed for CD34.

#### Other biopsies

In October 2009, the patient underwent a pleural biopsy along with a pericardial biopsy, which was conducted due to a large pericardial effusion. The pericardial biopsy showed a thrombogenic vasculopathy with ischemic necrosis accompanied by extensive vascular deposition of C5b-9.

## Discussion

We have presented a case of MAP in which the patient received biweekly eculizumab infusions commencing in October 2009. We have followed the patient’s clinical course and as well performed sequential biopsies over a 2.5-year period, monitoring the effects of the drug on his disease process from both a clinical and pathological perspective. As already alluded to, there are two aspects to the vascular injury in MAP, one of which is likely preferentially targeted by eculizumab. The first is characterized by a thrombotic microangiopathy affecting capillaries and venules whereby there is discernible endothelial cell injury with endothelial cell detachment and resultant vascular thrombosis. This microangiopathy is particularly apparent in active skin lesions although it also plays a role in the pathogenesis of the ischemic complications seen in other organ sites such as the brain, gastrointestinal tract and pericardium [1-3]. In addition part of the larger vessel pathology is one reflective of a similar pattern of endothelial cell injury and thrombosis.

The drug has almost an immediate effect on ameliorating small and larger vessel endothelial cell injury and vascular thrombosis. These effects were morphologically apparent after two doses of the drug, recognizing that the symptoms that accompanied the catastrophic thrombotic microangiopathy were improved significantly with the first infusion of the drug. We postulate that the basis is likely reflective of abrogation of endothelial cell apoptosis. While caspase 3, an apoptosis marker, was discernible within the endothelium of pre-eculizumab biopsies, a significant decrement in caspase 3 expression was observed within a few weeks of receiving the drug. There is literature precedent regarding C5b-9 mediated endothelial cell apoptosis primarily in the context of experimental models of glomerulonephritis [10]. We postulate that the deposition of C5b-9 likely reflected complement activation triggered directly by type I IFNs. There are a numbers of papers that suggest that IFNα, the main type I IFN, may be a direct catalyst to the activation of complement and eventually to C5b-9 deposition [11-15]. The high type I IFN signature was revealed by the extensive staining of MxA within endothelium and perivascular and interstitial inflammatory cells.

A point worthy of mention concerns the persistence of C5b-9 deposition for many months after the commencement of the drug, reflecting the slow clearance of C5b-9 previously deposited in affected tissues. Hence monitoring C5b-9 deposition in tissue is likely not of great value in assessing treatment response. From a morphologic perspective, post-eculizumab skin biopsies did not show active endothelial cell injury and/or thrombosis although more chronic change presumably on the basis of prior episodes of thrombotic microvascular injury may be quite impressive. Such changes include epidermal thinning, subepidermal fibrosis with vascular drop out, compensatory vascular ectasia and vascular basement membrane zone thickening.

In all of the biopsies, prominent MxA staining was noted in endothelium, perivascular and interstitial inflammatory cells, and vascular smooth muscle. The excessive type I IFN signature was not affected by eculizumab administration. Activated macrophages exhibiting erythrocyte phagocytosis along with mucin deposition persisted despite complement blockade, suggesting that its basis is a direct sequela of IFNα production. However, the downstream events of frank endothelial cell apoptosis and thrombosis within the vasculature appears to be halted by the drug.

Our patient continued to have significant gastrointestinal disease including peritonitis and bowel perforations despite receiving biweekly infusions of the drug. In gastrointestinal tract specimens procured 4months and 2 years after commencing the drug, there was persistence of the fibromyxomatous intimal arteriopathy although without vascular thrombosis. The segments of gut were resected due to perforation. We hypothesized that the basis of the perforation was one reflective of chronic and profound ischemia due to progressive occlusive nonthrombotic fibrointimal arterial changes. The lack of overt small and or larger vessel arterial thrombosis likely accounts for the efficacy of the drug in subverting catastrophic and likely fatal ischemic events. However intimal atherosclerotic like intimal narrowing although not specifically catastrophic can lead to chronic nonfatal ischemic changes analogous to the nonocclusive atherosclerotic narrowing operational in chronic ischemic syndromes such as angina pectoris and intermittent claudication. In this regard one might surmise the drug may reduce mortality but the long term morbidity of the disease remains. Currently our patient is very unwell and is progressively wasting due to persistent and likely progressive gastrointestinal disease.

There is now an emerging body of literature on the role of type I IFN in the evolution of the atherosclerotic-like larger vessel fibrointimal changes observed in patients with systemic lupus erythematosus [8,9]. Patients with lupus erythematosus display transitional upregulation of the IFNα/interleukin-18 (IL-18) processing machinery (a.k.a. the inflammasome) [16]. IL-18 has an inhibitory effect on endothelial cell differentiation, leading to decreased vascular repair with enhanced large vessel vascular injury Elevated levels of IL-18 also correlate with increased intima and medial wall thickness [17,18]. It is possible that the arteriopathy of MAP disease is pathogenetically very similar to the larger vessel arteriopathy of systemic lupus erythematosus whereby pathways independent of complement activation may be operational [18]. A finding that is particularly intriguing in our case was the massive infiltration of his gastrointestinal tract by CD14+ monocytes which exhibited an unusual pattern of arteriocentricity including their presence in the intima of diseased vessels. Further characterization of the proliferative intima revealed marked staining for smooth muscle actin although in the absence of staining for myogenin and desmin. The phenotypic profile of the intima was one compatible therefore with spindled cells showing CD14 positivity along with myofibroblastic differentiation. It is well established that CD14+ monocytes exhibit considerable plasticity and can undergo myofibroblastic differentiation [19]. At least in this one case, the source of the intimal myofibroblast, the putative cell responsible for the hyperplastic intima, could ultimately be derived from the surrounding CD14+ monocytes. The excessive number of CD14+ monocytes in the gastrointestinal tract may reflect enhancement of monocyte recruitment triggered by type I interferons. In addition under the influence of type I interferons, CD14+ monocytes show enhanced expression of sialic acid binding IgG lectin-1 binding (Siglec-1). Increased numbers of Siglec-1+ CD14+ monocytes has been implicated in systemic sclerosis associated pulmonary hypertension, systemic sclerosis [20,21], and coronary artery disease [22].

## Conclusions

The drug eculizumab aborts the severe catastrophic thrombotic microangiopathy events associated with Degos disease, likely through antiapoptotic endothelial cell effects. In this regard, the extent of thrombotic microangiopathic and larger vessel alterations are significantly attenuated although other deleterious sequelae associated with a type I interferon rich microenvironment such as endothelial repair dysfunction and enhancement of CD14+ monocyte influx with transdifferentiation into intimal myofibroblasts remain unaltered.

## Competing interests

The authors declare that they have no competing interests.

## Authors’ contributions

CMM was involved in the design of the study, interpretation and photographing of the biopsy material, and drafting and editing the manuscript. All of the other authors were involved in editing the manuscript and as well the patient was under the care of FB and MD; JL and LS provided invaluable input regarding therapy. All authors read and approved the final manuscript.
